# Discovery of new AMR drugs targeting modulators of antimicrobial activity using in vivo silkworm screening systems

**DOI:** 10.1038/s41429-024-00788-2

**Published:** 2024-11-14

**Authors:** Fumiaki Tabuchi, Kazuhiro Mikami, Masanobu Miyauchi, Kazuhisa Sekimizu, Atsushi Miyashita

**Affiliations:** 1 Teikyo University Institute of Medical Mycology, Hachioji, Tokyo Japan; 2 Graduate School of Medical Care and Technology, Teikyo University, Itabashi, Tokyo Japan; 3 Faculty of Pharma-Science, Teikyo University, Itabashi, Tokyo Japan

**Keywords:** Phenotypic screening, Medical research

## Abstract

Global concerns about drug-resistant bacteria have underscored the need for new antimicrobial drugs. Emerging strategies in drug discovery include considering the third factors that influence drug activity. These factors include host-derived elements, adjuvants, and drug combinations, which are crucial in regulating antimicrobial efficacy. Traditional in vivo assessments have relied on animal models to study drug absorption, distribution, metabolism, excretion, and toxicity (ADMET). Alternative models, such as silkworms, are being explored to overcome the ethical and financial barriers associated with mammalian models. The silkworm has been proven effective in evaluating ADMET and in highlighting the therapeutic potential enhanced by third factors. Host factors (either mammalian or non-mammalian) enhance the antimicrobial activity of antimicrobial agents such as lysocin E. Additionally, using d-cycloserine to potentiate vancomycin has successfully combated vancomycin-resistant infections in silkworms. Leveraging silkworms in drug discovery could establish a novel screening method incorporating interactions with third factors, whether host related or non-host-related, thus promising new pathways for identifying antimicrobial drugs with unique mechanisms of action.

## Introduction

Currently, the spread of pathogens resistant to multiple drugs, known as Antimicrobial Resistance (AMR), poses a global clinical challenge and has become a significant societal issue [1]. Examples of such drug-resistant bacteria include Methicillin-resistant *Staphylococcus aureus* (MRSA), Multidrug-resistant *Pseudomonas aeruginosa* (MDRP), and the pathogenic fungus *Candida auris*. Alongside the proliferation of these drug-resistant pathogens, there is a concerning trend towards a decrease in the number of new antimicrobial drugs reaching the market [1, 2], raising fears of a depletion of effective antimicrobial treatments available for clinical use. Therefore, there is an urgent need to develop drugs with new mechanisms of action. To address this challenge, accelerating infectious disease drug discovery through novel approaches different from traditional methods is crucial [1–3]. Enhancing the efficiency of exploratory research, particularly in the initial phase of drug discovery, is essential to prepare for the emergence of new drug-resistant bacteria and to expand the foundation for generating a diverse array of seed compounds. This review focuses on the development of drugs targeting factors that regulate the activity of antimicrobials, with the aim of overcoming infections caused by drug-resistant bacteria.

## Traditional antimicrobial drug discovery strategies and their limitations

Recently, in addition to the “bilateral interaction” between pathogens and antimicrobial agents, the existence of a third factor that synergistically interacts with antimicrobials has been highlighted [4].

The third factor in this context is defined as a substance other than the antimicrobial agent and its target, which influences the therapeutic effect of the antimicrobial agent. Incorporating this third factor into the traditional bilateral interaction is expected to pave the way for new discoveries in infectious disease drug development; however, the lack of sufficient knowledge regarding this concept, apart from a few attempts (e.g., see [5]), remains a challenge.

One of the challenges in infectious disease drug discovery based on bilateral interactions is that many seed compounds identified in exploratory research demonstrate antimicrobial activity in vitro but fail to show therapeutic efficacy in vivo. Mechanisms contributing to this discrepancy traditionally include factors such as absorption, distribution, metabolism, and excretion within the body, and potential toxicity [6, 7]. Therefore, it is crucial to gather information on pharmacokinetics and toxicity early in the exploration phase to identify promising compounds. In recent years, the significance of substances that interact with antimicrobials to enhance their therapeutic activity has grown. Examples of such third factors include apolipoprotein A-I, which can influence antimicrobial activity [4]. Failure to consider these factors during screening may lead to overlooking potential antimicrobial agents. Moreover, the requirement for candidate compounds to have low toxicity in vivo means that traditional screening methods based solely on the interaction between pathogens and drugs are inefficient in discovering effective drugs.

## Combination antimicrobial drugs

**Fig. 1:**
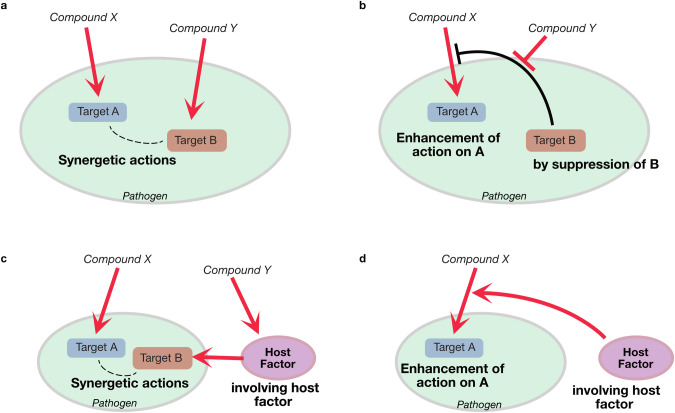
Examples and Mechanisms of Interactions with Antimicrobial Agents. These figures illustrate the mechanisms of interactions between antimicrobial agents and other factors. The diagrams are divided into four sections, each detailing different examples, and mechanisms of interaction. **a** This section shows the interactions between antibiotics with different mechanisms of action. This includes the effects of combining antibiotics that inhibit cell wall synthesis with those that inhibit protein synthesis. **b** This section explains the effects of combining efflux pump inhibitors or inhibitors of antibiotic-degrading enzymes with antimicrobial agents. Although metabolic inhibitors themselves do not exhibit antimicrobial activity, their combination with antimicrobial agents can enhance the antimicrobial effects. **c** This section demonstrates the interactions between host activation by immune stimulants and antimicrobial agents. It explains the effects of combining immune-activating factors, such as antimicrobial peptides, with antimicrobial agents. **d** This section shows the interactions between intrinsic factors and antimicrobial agents. It explains how endogenous factors can enhance the effects of antimicrobial agents

Antibiotics such as penicillin and streptomycin were administered as single agents at the time of their development. However, antibiotics are now given in combination to enhance antibacterial activity and combat the emergence of drug-resistant bacteria. The presence of combination drugs also affects the action of antimicrobial agents. The combination of sulfamethoxazole and trimethoprim is a typical antimicrobial combination, which is known to act synergistically in terms of antimicrobial activity [8]. The simultaneous administration of sulfamethoxazole and trimethoprim, which inhibit different enzymes in the folic acid synthesis pathway, is known to synergistically exhibit antimicrobial activity against pathogenic bacteria [9]. Moreover, the combination of amoxicillin and gentamicin has been shown to have a synergistic therapeutic effect on mice infected with penicillin-resistant *Streptococcus pneumoniae*, making the combination of antimicrobial drugs an important strategy for overcoming drug-resistant bacterial infections [10]. Additionally, colistin, known for disrupting cell membranes, acts synergistically with various antibiotics against colistin-resistant *Enterobacteriaceae* species [11]. A model diagram of the molecular mechanism of the interaction between the antimicrobial agent and the other factor described is shown (Fig. 1a & Table 1).

**Table 1:** Examples of interactions and molecular mechanisms between antimicrobial agents and other factors

Description of combination of antibiotic interactions	Combinations of compounds	Targets	Ref
The interactions through the combined use of antibiotics with different mechanisms of action (see Fig. 1a).	Amoxicillin + gentamicin	Cell wall synthesis (Penicillin-binding proteins (PBPs)) & protein synthesis (30S ribosomal subunit)	[10]
	Colistin + linezolid, rifampin, azithromycin, minocycline, clindamycin, erythromycin	Cell wall components (Lipopolysaccharides) & protein (30S or 50S ribosomal subunit) or RNA synthesis (RNA polymerase)	[11]
The interaction between metabolic inhibitors of pathogen metabolism or metabolites and antimicrobial agents (see Fig. 1b).	Amoxicillin + clavulanic acid	Cell wall synthesis (PBPs) & β-lactamase enzymes	[14]
	Gentamicin + glycerol monolaurate	Protein synthesis (30S ribosomal subunit) & Cell membrane	[15]
	Minocycline + loperamide	Protein synthesis (30S ribosomal subunit) & Cell membrane	[16]
	Tobramycin, gentamicin, kanamycin, paromomycin, streptomycin + inhibitors of *amg* function	Protein synthesis (30S ribosomal subunit) & Two-component signaling system (AmgRS system)	[17]
	Norfloxacin, Ciprofloxacin + efflux pump inhibitors	DNA replication (DNA gyrase and topoisomerase IV) & Drug efflux pumps	[18]
The interactions between host activation by immune stimulants and antimicrobial agents (see Fig. 1c).	Antimicrobial + streptazolin	Antimicrobial targets (depending on the agent) & host’s immune response	[19]
	AMPs (LL-37 and β-defensin 3) + Antimicrobial (tigecycline, moxifloxacin, piperacillin, meropenem)	Cell membranes and host’s immune response & DNA replication (DNA gyrase and topoisomerase IV) or protein synthesis (30S ribosomal subunit), cell wall synthesis (PBPs)	[20]
The interactions between endogenous factors and antimicrobial agents (see Fig. 1d).	Lysocin E, apolipoprotein A-1	Cell membrane components (menaquinone) & lysocin E	[4]

## Factors influencing the activity of antimicrobial drugs

### Host endogenous factors

One interaction factor that affects the activity of antimicrobial drugs is host endogenous factors. Certain antimicrobial drugs have been suggested to have capability of interacting with host molecules, leading to enhancement of their antimicrobial efficacy. For example, apolipoprotein A-I interacting with lysocin E [4, 12], enhances the antimicrobial efficacy of lysocin E. The therapeutic activity of lysocin E in in vivo experiments in mice infected with *S. aureus* was found to be higher than expected from in vitro experiments using the micro dilution method. This led to the hypothesis that host substances could enhance the activity of lysocin E. Upon adding either silkworm or various mammalian sera to in vitro experiments, the MIC value of lysocin E against *S. aureus* decreased, indicating an increase in antimicrobial activity. Apolipoprotein A-I was then purified from bovine serum as a molecule that enhances the activity of lysocin E. Although not identified by biochemical purification, lipid transport proteins (apolipophorin) similar to mammalian apolipoprotein A-I are conserved in silkworms. These results suggest that the host endogenous factors, either apolipoprotein A-I in mammals or the unidentified molucules in the silkworm, can increase the antimicrobial activity of lysocin E (Fig. 1d, Table 1).

### Antimicrobial adjuvants

Another group of compounds that influence the action of antimicrobial drugs is known as antimicrobial adjuvants [13]. These compounds do not possess antimicrobial activity themselves but can enhance the effectiveness of antimicrobial drugs when used in combination. One example of antimicrobial adjuvants includes β-lactamase inhibitors like clavulanic acid, which are alongside β-lactam drugs such as amoxicillin. β-lactamase inhibitors are used to enhance the action of the co-administered antimicrobial drug (e. g., amoxicillin) by inhibiting antibiotic-degrading enzymes in β-lactamase positive, β-lactam-resistant bacteria [14]. Additionally, surfactants and cell membrane disruptors, such as glycerol monolaurate, are known to increase antimicrobial activity by enhancing the permeability of antimicrobial drugs to pathogens [13]. For instance, adding glycerol monolaurate has been shown to increase the antimicrobial activity of gentamicin against *S. aureus* in biofilms [15]. Loperamide, used as an antidiarrheal, has been experimentally confirmed to enhance the uptake of the antimicrobial drug minocycline into pathogens by affecting the proton motive force of pathogenic bacteria, resulting in an increased pH gradient across the bacterial cell membrane [16]. In addition, transposon mutants of the bacterial two-component signaling system genes *amgR* and *amgS* have been shown to exhibit increased sensitivity to antimicrobial agents [17]. However, substances that specifically inhibit the functions of these genes have not yet been reported. Therefore, the compounds that inhibit the functions of these genes could serve as adjuvants that enhance the antibacterial effects of antimicrobial agents when used in combination. Furthermore, compounds such as flavonoids (e.g., genistein and sarothrin), which have been shown to inhibit the activity of a drug efflux pump, are expected as antibacterial adjuvants [18] (Fig. 1b & Table 1). Streptazolin, considered as an enhancer that boosts the host’s immune capability, is also a potential candidate for antimicrobial adjuvants [19]. Furthermore, recent studies have shown that the combination of immune-activating agents with antibiotics can significantly enhance antimicrobial efficacy. Antimicrobial peptides (AMPs) such as LL-37 and β-defensins modulate the host’s innate immune response, thereby enhancing pathogen clearance [20]. By disrupting bacterial membranes and promoting the recruitment of immune cells, these agents facilitate the penetration and efficacy of antibiotics, particularly against resistant strains (Fig. 1c & Table 1).

*Galleria mellonella* has been used to evaluate interactions between antimicrobial agents and antimicrobial adjuvants [21]. In this study, the synergistic effects of oxethazaine, a compound found in in vitro experiments, and colistin on Gram-negative pathogens were evaluated in in vivo assay systems using the mouse sepsis model and *Galleria mellonella*. In addition to this, the synergistic effect of colistin and levofloxacin was also investigated [22]. The silkworm model, also using insects, may be useful as an in vivo discovery system for such antimicrobial adjuvants.

## Attempts at drug discovery research using silkworms

We propose the utilization of a drug discovery platform using silkworms as infection hosts from the early stages of screening to accelerate the development of infection control methodologies [4, 6, 23–34]. The advantages of using a silkworm model for in vivo experimental systems include the following:

Firstly, silkworms have a drug metabolism mechanism similar to mammals, and it has been found that the ED_50_ values of various therapeutic drugs for silkworms infected with pathogens correspond well with mammalian models [24, 35]. Moreover, due to the low cost of rearing and the capability to rear large numbers of individuals at once, silkworms can be an alternative to in vitro screening systems.

Additionally, in developed countries, there is increasing regulation on the use of mammals in animal experiments from an animal welfare perspective [36], leading to a strong demand for alternative animal models to mammals.

Given these advantages, the use of the silkworm infection model is expected to accelerate drug screening that takes into account the presence of a third factor including the host factors. In this article, we will discuss specific examples of factors that interact with antimicrobial drugs and argue that combining the silkworm infection model with the third factor in a tripartite interaction can make the discovery of new antimicrobial drugs more efficient.

**Fig. 2:**
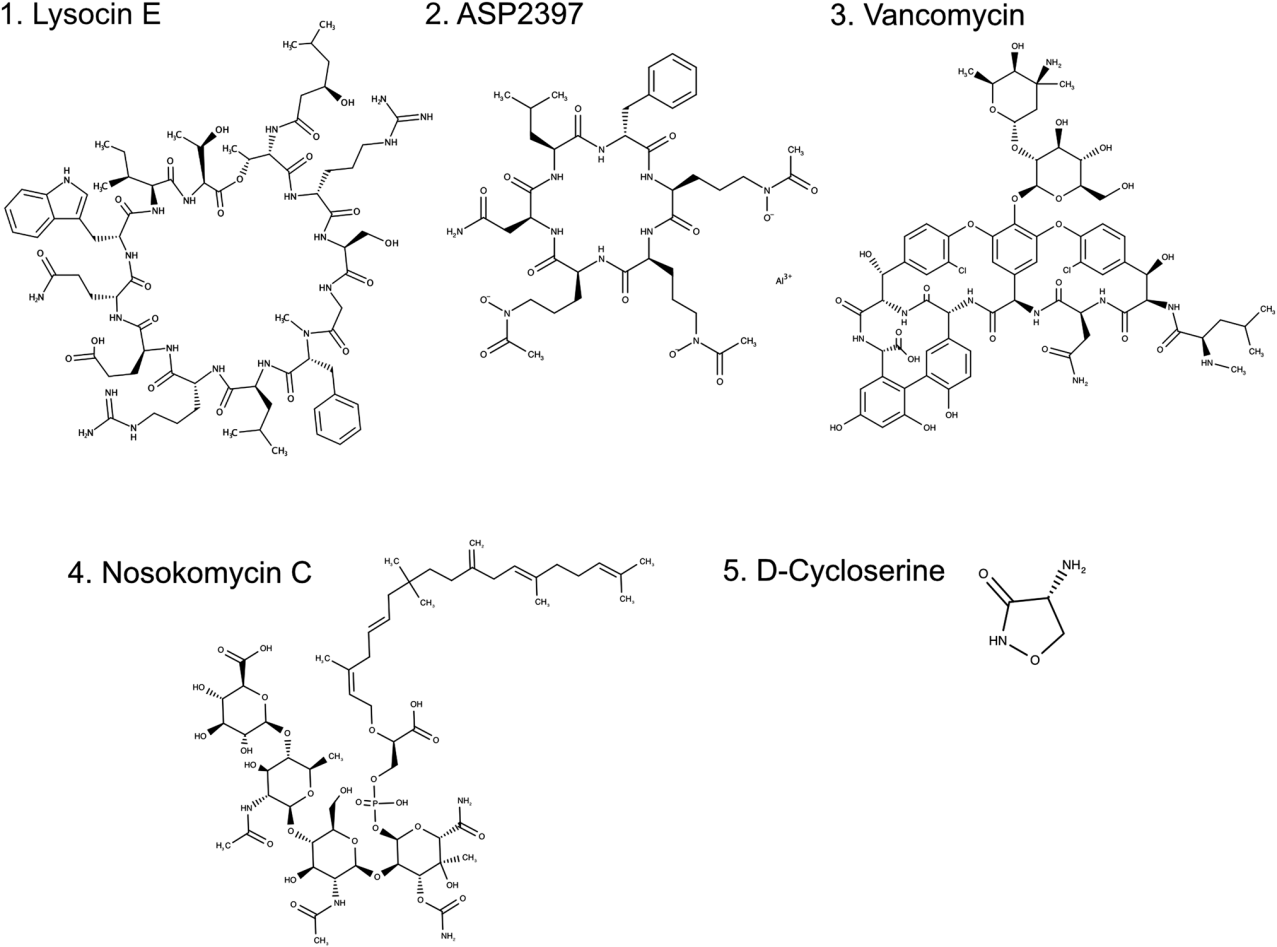
Chemical structures of compounds discussed in this paper. In this figure, the chemical structures of compounds discussed in this paper are shown. 1. Lysocin E, 2. ASP2397, 3. Vancomycin, 4. Nosokomycin C, and 5. D-Cycloserine

In addition to lysocin E, previously mentioned, nosokomycins (nosokomycin A-D; see Fig. 2 for chemical structure of nosokomycin C), discovered in 2010, are another example of antimicrobial drugs found using the conventional silkworm drug discovery platform [37]. Nosokomycins were identified as novel antimicrobial agents through in vitro screening and screening using the silkworm model from a library of 5340 microbial strains (actinomycetes, fungi) and have been shown to exhibit excellent therapeutic activity against MRSA infections [38, 39]. Nosokomycins, due to their structural similarity to previously studied moenomycin A [37, 40], are presumed to exhibit antimicrobial activity by inhibiting bacterial cell wall synthesis through the inhibition of peptidoglycan glycosyltransferase (PGT) [41].

Furthermore, from a library of 310 fungal strains showing anti-*Aspergillus* activity, ASP2397 (refer to Fig. 2 for chemical structure) was discovered through screening using the silkworm model and is anticipated as a novel antifungal [28, 42, 43]. ASP2397 demonstrated superior therapeutic activity in a mouse model of pulmonary aspergillosis in vivo assays, even when compared to existing drugs [43]. ASP2397, with its siderophore-like structure, is believed to be taken up by fungi through siderophore-specific transporters and exhibit antifungal activity [43].

Among the examples shown above, lysocin E, in particular, has been shown to act synergistically with a host factor, apolipoprotein A-I, a third factor defined in this paper, to enhance therapeutic activity against *S. aureus* infection [4]. It is known that insects, including silkworms, contain apolipophorin, a lipid transport protein like apolipoprotein (as mentioned in the preceding section). Although no endogenous host factors have been identified in silkworms that enhance the antimicrobial activity of lysocin E, it is anticipated that such lipid transport proteins may enhance the antimicrobial activity of lysocin E as apolipoprotein A-I does [4]. Therefore, it is expected that the silkworm-based screening system will enhance the new drug discovery considering the third factors.

Currently, there are several challenges that remain unresolved in silkworm models for drug discovery. For instance, compared to mammalian models such as mice, there is a need for additional evidence to extrapolate results to humans. In addition, the silkworm model has not been established for the evaluation of infectious agents such as skin infections and other topical agents. Furthermore, the silkworm model has yet to explore therapeutic agents for highly host-specific RNA viruses and parasites such as *Plasmodium falciparum* malaria. Overcoming these challenges will pave the way for a new silkworm-based drug discovery platform that integrates third factors.

## Potential of the silkworm model beyond pharmacokinetics and toxicity profiles: drug discovery focused on interaction factors

### Future perspective

Certain antimicrobial agents are known to act synergistically against infectious agents when administered in combination, while at the same time potentiating the side effects of the antimicrobial agents. Vancomycin, for example, is known to increase reactive oxygen species (ROS), which is directly toxic to renal cells, causing side effects such as renal failure [44]. When used in combination, vancomycin and aminoglycosides such as gentamicin are known to increase the risk of renal failure [45]. It has also been suggested that the combination of the beta-lactam agents piperacillin and vancomycin may increase the risk of acute kidney injury [46]. Other studies have shown that the combination of beta-lactams and beta-lactamase inhibitors may enhance gastrointestinal symptoms such as diarrhea and liver dysfunction [47].

In general, the combination of antimicrobials is expected to reduce the concentrations of individual drugs and the incidence of side effects. However, as mentioned above, combined use of several antimicrobial agents may potentially synergistically increase the toxicity risk. Therefore, the toxicity of drug combinations should be carefully evaluated for clinical practice. For 59 drug compounds, it has been shown that acute lethality (LD_50_) measured in the silkworm model correlates well with that measured in mammalian models, indicating the usefulness of the silkworm model as an alternative model for evaluating the toxicity of individual drug compounds [48]. Furthermore, studies have shown that silkworms possess several leakage enzymes, such as alanine aminotransferase (ALT) and lactate dehydrogenase (LDH), which enables biomarker-based sublethal toxicity prediction as in mammalian systems [49, 50]. However, there is limited knowledge regarding the potential synergy in toxic effects when combined use of drugs, necessitating further research to establish toxicity evaluation for drug combination in the silkworm model.

### Drug discovery indexed by the enhancement of antimicrobial activity through combination drugs

Using a screening method with silkworms infected with vancomycin-highly resistant MRSA, Tabuchi and colleagues found ceftriaxone, a β-lactam drug, acts synergistically with vancomycin (see Fig. 2 for chemical structure) and enhances therapeutic effects against silkworms infected with vancomycin-resistant MRSA [51, 52]. Although oxacillin also showed synergistic effects with vancomycin in in vitro micro dilution tests, it did not increase therapeutic activity in silkworms infected with vancomycin-resistant MRSA [51, 52]. This difference is likely due to the half-life of the drugs in the body: oxacillin has a short half-life of 0.5 h in mammals [53], whereas ceftriaxone’s is 6–9 h [54]. This longer half-life allows ceftriaxone to co-exist with vancomycin (half-life of 4–8 h) in the bloodstream for an extended period [55], thereby facilitating a stronger synergistic effect in the silkworm model. While there is no direct information on the half-life of oxacillin and ceftriaxone in silkworms, it is known that silkworms possess drug-metabolizing enzymes such as P450s and enzymes involved in conjugation reactions, and that some pathways in metabolism and excretion are shared with those in mammals [30, 35]. Therefore, it is inferred that differences in drug localization within the silkworm body may lead to variations in synergistic effects for treating infections. These results suggest that in vivo experiments using silkworms can enable the screening of combination drugs, taking into account the excretion and half-life of drugs within the body.

Additionally, Tabuchi and colleagues screened a variety of antimicrobial drugs with different mechanisms of action for synergistic effects with vancomycin against vancomycin-resistant *S. aureus*. They discovered that d-cycloserine (see Fig. 2 for chemical structure) sensitizes all eight tested vancomycin-resistant MRSA strains to vancomycin when used at a 1/4 MIC concentration. Furthermore, when silkworms infected with vancomycin-resistant MRSA were treated with a combination of vancomycin and d-cycloserine, there was a significant increase in survival compared to either drug alone [56]. At present, the combination effect of d-cycloserine and vancomycin has only been observed in the silkworm model, and the concept has yet to be established. Further research in mammalian models, such as mice, will be necessary to evaluate potential applications to human medicine.

By utilizing similar methods, future searches for new antimicrobial agents that enhance therapeutic activity in combination with existing antimicrobials, whether from natural products, compound libraries, or previously discontinued drugs, are expected to yield effective treatment options against drug-resistant bacteria.

### A case study: lysocin E

Lysocin E (see Fig. 2 for chemical structure) targets menaquinone molecules on bacterial cell membranes and is believed to exhibit antimicrobial activity by causing cell membrane disruption [12]. Further research revealed that apolipoprotein A-I in the blood promotes the cell membrane disruption activity of lysocin E [4]. Lysosin E interacts with lipid-II, a precursor molecule of cell wall syntheses present in the cell membrane [57], and furthermore, lipid-II has been shown to interact with apolipoprotein A-I. Given these facts, it is speculated that the interaction with lipid-II facilitates the binding of lysocin E and apolipoprotein A-I, significantly increasing the activity of lysocin E in destroying the cell membranes of pathogenic bacteria [4, 12, 58]. Traditional in vitro screenings, which only consider the bilateral interactions between pathogens and drugs, may overlook candidates that exhibit antimicrobial efficacy potentiated by interaction with host endogenous factors. Hence, incorporating host-derived third factors will facilitate the discovery of antimicrobial drugs with novel structures or mechanisms of action.

## Conclusion

**Fig. 3:**
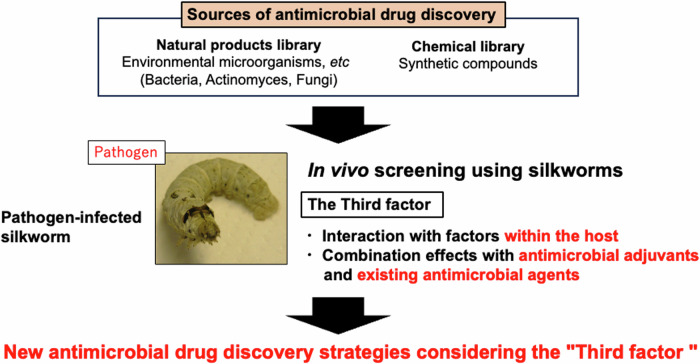
Silkworm-based novel AMR drug discovery focusing on modulators of antimicrobial activity. This figure illustrates an overview of strategies for the creation of novel antibiotics and antifungal agents from resources such as natural product and compound libraries, utilizing an in vivo silkworm-based screening system

Utilizing in vivo screening systems with silkworms enables efficient discovery of novel antibiotics and antifungal drugs (Fig. 3). This approach facilitates the consideration of “third factors” including interactions with host endogenous factors, synergistic effects with antimicrobial adjuvants, and combination effects with existing antimicrobial drugs. By administering drug candidate samples obtained from natural product and compound libraries to pathogen-infected silkworms, a new strategy for antimicrobial drug discovery that accounts for these third factors can be realized more swiftly and efficiently.
